# The role of etiopathogenetic aspects in prediction and prevention of discontinuous-hemorrhagic (Mallory-Weiss) syndrome

**DOI:** 10.1186/s13167-016-0056-4

**Published:** 2016-03-20

**Authors:** Evgeny F. Cherednikov, Anatoly A. Kunin, Evgeny E. Cherednikov, Natalia S. Moiseeva

**Affiliations:** 1 Faculty of General Medicine, The Department of Faculty Surgery, Voronezh N.N. Burdenko State Medical University, Avenue of Revolution Str. 14, Voronezh, Russia; 2 Faculty of Dentistry, The Department of Faculty Dentistry, Voronezh N.N. Burdenko State Medical University, Avenue of Revolution Str. 14, Voronezh, Russia; 3 Streleckaja Bol’shaja, 20 B, 69, Voronezh, 394035 Russia

**Keywords:** Etiology, Pathogenesis, Discontinuous-hemorrhagic (Mallory-Weiss) syndrome, Cardioesophageal area, Preventive diagnostics, Gastroduodenal bleedings, Granular sorbents, Endoscopy, Prevention, Treatment

## Abstract

The article contains an overview of the literature on Mallory-Weiss syndrome. It analyzes numerous etiological factors, provides new insights into the pathogenesis of the disease, gives a description of a previously unknown dependence of discontinuous-hemorrhagic syndrome on the topographic and structural features of the cardioesophageal area of the digestive tract, and gives scientific credence to methods of prediction, prevention, and treatment of the syndrome with complex involvement of granular sorbents.

## Background

Discontinuous-hemorrhagic (Mallory-Weiss) syndrome is considered an emergency condition in abdominal surgery. If 30 years ago, bleedings resulting from discontinuous-hemorrhagic syndrome were rare, they are now prevalent among all bleedings of non-ulcer genesis [1–6].

Despite significant advances in endoscopic treatment of gastroduodenal bleedings, the hemorrhage recurrence rate in discontinuous-hemorrhagic syndrome reaches 20–30 %, postoperative mortality—10–17 % [4, 7–9], and overall mortality—7.5–8.6 % [10].

## Etiology of discontinuous-hemorrhagic (Mallory-Weiss) syndrome

The American scientists Mallory and Weiss, in 1929 and 1932, respectively, revealed the cause of upper gastrointestinal bleedings: mucosal tears at the junction of the stomach and esophagus. This syndrome was associated with recurrent vomiting after drinking and a heavy meal [11, 12].

According to Mearin et al., the effect of alcohol on the occurrence of bleeding tears in the cardioesophageal area is that it acts as a factor directly or indirectly causing vomiting. In addition, ethyl alcohol affects the esophageal and gastric mucosa by increasing hydrogen ions retrodiffusion, which reduces its protective properties. And thirdly, alcohol intoxication may disrupt the esophageal motor activity and pressure in the lower esophageal sphincter [13].

The literature contains data on other etiological factors that contribute to the syndrome development. They are primarily the reasons of a rapid increase in intra-abdominal and intra-gastric pressures: vomiting of different origin, blunt abdominal trauma, severe coughing, epilepsy, acute severe asthma, constipation, delivery, closed-chest cardiac massage, lifting weights, endoscopic examination, and emetic reflex which can occur in patients during dental treatment [8, 14–19].

## Role of the emetic reflex in discontinuous-hemorrhagic (Mallory-Weiss) syndrome

The emetic reflex is triggered on the back wall of the throat. It prevents from intrusion of foreign objects into the respiratory ways. Some people have a very sensitive gag reflex, and its coverage is extended up to the front teeth. This causes certain inconveniences during a dental visit such as difficulties in X-ray imaging, tooth pattern contouring, using of a saliva ejector, treatment of the lateral teeth, or wearing of removable dentures.

There are two types of reactions causing the emetic reflex: physiological reaction—a response reaction to mechanical stimulation, a contact in the mouth; and psychological reaction—a reaction connected with a stomatophobia. The combination and mutual influence of both types of reactions is more frequent.

The emetic reflex may become more intensive in connection with labored nasal breathing, stomach disorders as well as with pregnancy. The memory of unsuccessful treatment experience can form a constant fear and evoke the reflex at the sight of tools, with a smell of medications, or a sound of the equipment.

In different people, the reflex intensity varies from slightly pronounced to very severe.

The effective means for a mild intensity can be as follows:

- Using of a cofferdam when the oral cavity is isolated from an affected tooth by a rubber sheet and there is no danger of accidental contacts between the instruments and the palate or gums
- Psychological distraction techniques (music, TV), relaxations
- Superficial anesthesia
- Using of antiemetic agents on the eve and 1 h before a dental visit
- Stage-by-stage retraining that can be successfully done at home and relieve from the painful reflex for good

The effective means for a medium intensity are as follows:

- Dental treatment using nitrous oxide sedation combined with further treatment of a stomatophobia helps many patients significantly. Some doctors fall back on anesthesia by application of lidocaine followed by bilateral anesthesia of a lingual nerve *to remove* the reflex.

The effective means for a severe intensity can be the following:

- When the reflex is extremely intense and other approaches are not effective, the teeth are treated under general anesthesia or intravenous sedation. In this case, the patient feels nothing, which makes it possible to carry out the whole list of necessary manipulations. However, sedation and general anesthesia must be performed by a qualified anesthesiologist. Every clinic must be properly equipped for any complications.

There are a number of recommendations for this group of patients:

1. If you know about your tendency to the emetic reflex, do not eat food 2–3 h before a visit to the dentist.
2. Make an appointment with your dentist after the noon. It is noticed that the sensitivity of the reflex is increased in the morning hours.
3. The possibility to breathe through the nose will significantly reduce the reflex probability. Use nasal drops or solutions before a dental visit if necessary.
4. With the first symptoms of the reflex, breathe in rhythmically through the nose and breathe out through the mouth. As it is impossible to breathe and swallow simultaneously, so the emetic reflex cannot be triggered while breathing.
5. Try to hum some tunes. This will create a continuous air flow and will prevent the reflex.
6. The retraining or receiving dental care will be easier if there are some distractions during the procedure. So, listen to music, watch TV, or take ice in your hands. Salt water mouthwash rinses may be used to help some people.
7. The retraining is strongly recommended to those patients who use removable prosthetic appliances.
8. Learn how to relax. Talk to the dentist. Clear up all the questions that may cause your concern or anxiety. Tell the doctor about the treatment background, about the first time the reflex occurred, and about the things that evoke it. The confidential relations as well as the understanding of what is going on will make you feel comfortable and will help the doctor to avoid unpleasant procedures.

Another predisposing factor to the Mallory-Weiss syndrome, in some authors’ opinion, is sliding (axial) hiatal hernia (SAHH), which occurs in patients in 35–100 % of cases [5, 19–23]. Such considerable variation in data may be explained by a variety of diagnostic techniques for sliding (axial) hiatal hernia. Tutukov et al. [24] indicate that in case of upper gastrointestinal bleedings, patients should undergo targeted examinations to identify the hernia.

According to Cicia [25], the existence and sizes of sliding (axial) hiatal hernia have an impact both on the incidence and localization of tears. In SAHH, the internal pressure in the hernial cavity is identical to the intra-gastric pressure. At the same time, the forestomach wall is more affected by this positive pressure than by a negative intrathoracic one. As a result, in severe attacks of retching, the cardiac region of the stomach experiences great transmural pressure, so the tears that occur in patients with SAHH are observed to be localized in the stomach. The complete absence of SAHH is common in case the tears are localized in the esophagus. With their localization at the gastroesophageal junction, SAHH will be detected with great difficulty in the course of examination of a patient at rest but will only appear at the moment of a rapid rise in intra-abdominal pressure.

A favorable background for the development of tears is acute and chronic gastritis, gastroduodenal ulcer, acute pancreatitis, and esophagitis [14, 18, 19, 23, 24, 26, 27].

### Pathogenesis of discontinuous-hemorrhagic (Mallory-Weiss) syndrome

The pathogenesis of Mallory-Weiss Syndrome has not been fully studied by now. The authors have presented its mechanism as follows: in retching, the pylorus is closed, and the cardiac part of the stomach and the esophagus are dilated. The gastric contents, due to antiperistalsis and a sudden increase in intra-abdominal pressure, impetuously move forward to the gastroesophageal hole. The result is that intra-gastric pressure is rapidly increased, the cardiac part of the stomach is hyperextended, and its mucosa is lacerated [12].

Miroshnikov and Rasskazov believe that the process of formation of bleeding tears in the cardioesophageal area is a complex multifactorial mechanism, whose development is determined by a combination and interaction of several closely interconnected mechanisms. Most important among them are the following: (1) limitation of the mobility between mucosal and submucosal layers of the cardia; (2) discoordinated and oppositely directed contraction of differently oriented muscles in the lower third of the esophagus and cardia; and (3) insufficiency of the closing apparatus of the cardia. This combination of factors predisposes the mucosal tear formation. But their direct emergence is associated with a sudden change in intra-gastric pressure [19].

Bellmann and Wohlgemuth suggested an involvement of biophysical factors in the formation of mucosal tears along the lesser curvature of the stomach—the main point of intra-gastric pressure application in its rapid increase. In the authors’ opinion, linear lacerations appear due to the longitudinally arranged gastric folds in the cardioesophageal area. Having studied age-related changes in the submucosal layer, the authors revealed a loss in the collagen fibers strength increasing with age and leading to a limitation of mobility between the mucosal and submucosal tissues in this area and formation of tears in a poorly extendable mucosa in rapid increasing of intra-gastric pressure [28].

The anatomical structure of the lower third of the esophagus and cardia also plays an important role in the Mallory-Weiss Syndrome development. The relevance of morphological studies in this area has been confirmed by many scientists [1, 6, 19, 28–33].

Cherednikov et al. have done theoretical multifunctional, morphological, biophysical, experimental, and clinical research, which made it possible to reveal a new, previously unknown pattern of the development of discontinuous-hemorrhagic (Mallory-Weiss) syndrome recognized as a discovery. The pattern consists in formation of vertical bleeding tears at the gastroesophageal junction with their predominant localization on its right and back walls, which is associated with different thickness of the digestive tract tissues [1, 29, 31, 34].

Cherednikov, Batkaev et al. and Bratus et al. have proven that the stretching force *F* affecting the gastroesophageal junction walls in a sudden increase of intra-gastric pressure (in vomiting) equals to 110.3 kg. It has been also noted that the emergence of discontinuous-hemorrhagic syndrome greatly depends on anatomical differences in the structure of the gastroesophageal walls in the cardioesophageal area, features of the histological structure of the tissues, and characteristics of the ligaments of the area, which taken as a whole determine different values of the elastic properties of the relevant sections of the gastroesophageal junction [35–37].

The left and front cardioesophageal parts are unbound because, unlike the right and back sections, they are not fixed with the ligaments. The force acting under the influence of excessive pressure in the direction of the front left semicircle of the gastroesophageal junction is balanced out by the quasi-elastic force F1. There is a critical value of the quasi-elastic force *F* equal to the value of the stretching force F1, in which there is a tear in the different layers of the gastroesophageal junction wall, when a further increase of the force F1 is impossible due to the limited elasticity of the tissues. Moreover, a tear in the left section (with a thinned wall) appears when the pressure is even less than in the front section with a larger amount of circular layers of the digestive tract.

According to the authors, anatomical arrangements and physical patterns are different in the right and back sections, which are fixed with the ligaments. In the direction of the right and back sections, the stretching force *F* is not spent on gas compression and is always greater than in the direction of the front and left parts, and the elastic properties of tissues in the right and back sections are low because of the ligamentous apparatus. Thus, with a sudden and consistent increase of pressure in the stomach, a tear is more likely to appear in the right and back sections, less often in the left (more flexible and mobile due to the presence of the gastric air bubble) section, and rarely in the front (most powerful and poorly fixed) section of the cardioesophageal area.

This discovery is of great practical importance for preventive diagnosis of Mallory-Weiss syndrome: it makes a physician to focus on the most frequent localization of tears, on the most dangerous and unstable, in terms of recurrent bleedings, right wall (a. gastrica sinistra) of the gastroesophageal junction. It is also important for the prediction of Mallory-Weiss syndrome severity with the aim to prevent errors and complications and for prescribing particular preventive measures with the aim to prevent recurrent hemorrhage [1, 6, 21, 38, 39].

Despite the large number of studies on the typical features of different organs, a research on the typical features of the stomach in patients with discontinuous-hemorrhagic syndrome and on a constitutional predisposition to the features of the clinical course of Mallory-Weiss Syndrome, which is relevant for clinical practice, has not been conducted.

The available literature describes the only survey carried out by Cherednikov with the aim to study the correlation between a patient’s constitution and the clinical course of Mallory-Weiss Syndrome [40].

Having conducted anthropometric studies in 23 patients with Mallory-Weiss syndrome, the author received previously unknown data on patients’ constitutional predisposition to some characteristics of the disease development. A constitutional type was studied according to V.N. Shevkunenko.

The following data were obtained in the course of research by Cherednikov [40]:

- Single tears at the gastroesophageal junction are more common in people of a brachymorphic type (average or below average height, long body, short legs, broad back, broad chest, considerable weight), and multiple tears are more common in individuals of a dolichomorphic type (tall or above average height, short body, long legs, narrow shoulders, long chest, small weight). Moreover, single tears occur 3.4 times more than multiple. All of them are vertical.
- Tears on the right and back walls are typical for patients with a distantia spinarum equal to 25.1 ± 0.44 cm (*p* < 0.05) and more, and tears on the left and front walls are typical for patients with a distantia spinarum equal to 22.1 ± 0.40 cm (*p* < 0.05) and less. With the chest circumference equal to 97.4 ± 1.42 cm (*p* < 0.05), tears are more frequent on the right wall; if it is 96.0 ± 1.35 cm (*p* < 0.05), on the back wall; and if it is 67.1 ± 1.30 cm (*p* < 0.05), on the left and front walls.
- Less height of the epigastrium—10.3 ± 0.44 cm (*p* < 0.05)—is more observed in patients with a continuous bleeding and the risk of its formation than in patients with a stanched bleeding—12.3 ± 0.39 cm (*p* < 0.05) and more.

Thus, the analysis of the data obtained by the author has shown that such available anthropometric studies as measurement of a distantia spinarum and epigastrium height may be useful in preventive diagnosis of tear localization and prediction of a course of bleeding in discontinuous-hemorrhagic syndrome. This may improve the diagnostic efficiency necessary to prevent errors and complications.

## Endoscopic techniques for Mallory-Weiss syndrome

It is known that there is no specific therapy for Mallory-Weiss syndrome but some therapeutical elements that are used to stop both ulcer and non-ulcer gastroduodenal bleedings may be applied.

Obviously, bleeding cardioesophageal tears have a higher, in comparison with chronic bleeding gastroduodenal ulcers, ability for hemostasis. The latter is characterized by the existence of an ulcer crater surrounded by dense rigid rims with a dissolute vessel in the center. As for tears, they, due to their linear form in soft, non-rigid walls, especially at early stages of their formation, tend to be closed. This is mainly associated with a purely mechanic deflation of walls, which contributes to the rapid development of its reparative processes. Such a tendency to spontaneous hemostasis is typical mostly for surface tears located within a mucous-submucous layer of the stomach wall. This explains most surgeons’ decision to adhere to conservative therapies for patients with Mallory-Weiss syndrome [1, 9, 10, 35, 41]. As for deep and wide tears reaching the muscular layer, they are in a greater degree characterized by the regularities common for chronic bleeding ulcers, namely a tendency to recurrent bleedings due to the fermentation of a blood clot caused by peptic or antiperistaltic (in repeated vomiting) movements of the gastric wall [16, 37]. Taking this fact into account, many authors focus on active conservative measures in order to find new endoscopic opportunities not only in the treatment of bleeding defects of the cardioesophageal zone but also, what is more important, in the prevention of recurrent bleedings [33, 35, 42–46].

Therapeutic endoscopy may appear as the only reasonable method of treatment for patients with Mallory-Weiss syndrome [1, 6, 8, 10, 33, 45, 47, 48].

All known methods of endoscopic hemostasis can be divided into thermal, injection, and application techniques.

## Thermal methods of endoscopic hemostasis

Thermal methods include monopolar and bipolar electrocoagulation, laser photocoagulation, cryocautery of the source of bleeding, and others [40, 42, 45, 49, 50].

Bondarenko reports on 41 patients with profuse continuous ulcer bleeding, who underwent hemostasis using a high-frequency coagulation device with a fluid electrolyte solution, the so-called liquid diathermocoagulation. Hemostasis carried out by means of liquid coagulation was achieved in all cases, while conventional monopolar coagulation failed to prevent bleeding in approximately 25 % of those cases. Coagulation by electrolytes is devoid of such negative properties as adhesion of the coagulated tissue to the electrode, followed by separation of a scab at the point of its removal from the coagulation zone. Deep coagulation is dangerous due to the risk of recurrent bleedings on 3–5 days in connection with the sloughing and exposure of blood vessels of the submucosal and deeper layers of the gastric and, especially, the intestinal walls. Direct coagulation of a bleeding vessel by a liquid coagulator occurs without separation of a scab adhered to the electrode; it is more sparing and effective [49].

However, the author describes the results basing on a small amount of clinical material and using this method only for ulcer hemorrhages. It seems promising to use the method by liquid diathermocoagulation for discontinuous-hemorrhagic syndrome.

## Injection methods of endoscopic hemostasis

Injection methods to stop gastroduodenal bleedings are widely used because of their availability and implementation simplicity: ε-aminocaproic acid, epinephrine, and ethanol injections. The initial hemostatic effect of injection methods is high and reaches 80.5–90 %. Herewith, the data given by the same authors show that recurrent bleedings reach 14.2–24.1 % [40, 42, 51–53].

## Application and insufflation techniques of endoscopic hemostasis

Another method to stop bleeding includes endoscopic adhesive applications with syringe barrels or cartridges: Lifusolum, Statisolum, Gastrosolum, biological adhesives MK-6, MK-8, and others. Parkhisenko injected a biological adhesive under pressure using a needleless injector, which provides reliable hemostasis by forming hemostatic infiltration in tissues and specific adhesive seals [54]. However, the adhesive compositions do not possess sorption properties but produce a water-repellent effect, film-forming polymers do not have local hemostatic properties, and moreover, ulcerous defects after being treated repair with rough scar formation [36, 54].

The method of endoscopic treatment of gastroduodenal ulcers by insufflation of dry powdered gel sorbent into a defect region has become widespread in recent years [36, 42, 55, 56].

Hydrophilic granular sorbents are polymeric agents capable to expand in aqueous solutions and form soft gels. Some authors [24, 36, 57] call them gel sorbents or hydrogels, and other researchers [33, 35, 49, 58–60] classify them as biologically active draining sorbents.

Most of these preparations are obtained synthetically by polymerization of molecules of dextran, polyvinyl alcohol, acrylamide, monomethacrylate, ethylene glycol. Dextran gels are represented by Sephadex, Cytodex, Molselect; acrylates and biogels of different types are acrylamide derivatives; monomethacrylate sorbents are represented by hydrogel; and polyvinyl alcohol derivative is Gelevinum [55, 58, 59, 61, 62].

Cherednikov created an absolutely new trend for the clinical use of Gelevinum and its derivatives in the field of gastroenterology: sorption-insufflation therapy for erosive and ulcerous lesions of the gastrointestinal tract. The proposed method of endoscopic pneumo-insufflation of Gelevinum has been proved to be effective in patients with chronic gastroduodenal ulcers, with acute gastroduodenal ulcers, with indolent peptic ulcers, and with gastroduodenal ulcers complicated by bleedings [36, 60].

Cherednikov explains the mechanism of action of Gelevinum for ulcerous bleedings as a multidirectional combined effect of the sorbent. On the surface of the Gelevinum pores, there are processes of adsorption and absorption of plasma proteins: fibrinogen, γ-globulin, etc., with their subsequent aggregation and cell elements adhesion to these proteinic layers. Gelevinum becomes a kind of component of the clot matrix, and, as a result, makes it less susceptible to the processes of retraction and the plasma system effects. On the other hand, as the author indicates, absorbing protein Gelevinum swells up and turns into a soft elastic gel layer with marked adhesive properties. Through this process, the period of detachment of the elastic gel layer from the ulcer surface is 5–7 days; it occurs gradually with the ulcer surface epithelization. Having performed its hemostatic “mission”, Gelevinum subsequently acts as an endoprotector and biological stimulator of the detritus-free ulcer surface, contributing to the growth of granulations and epithelial recovery [36, 56].

Batkaev writes about endoscopic application of another granular sorbent Diotevin with antimicrobial effects in 155 patients with non-ulcerous gastroduodenal bleedings [35].

As the authors note, hydrophilic granular sorbents have a complex and multidirectional action, generate a protective hydrogel layer resistant to gastric contents, possess non-specific hemostatic properties, have an anti-inflammatory effect, and create conditions for active reparative processes in the defective area. All this gives grounds to use hydrophilic granular sorbents for prevention and local treatment of discontinuous-hemorrhagic syndrome.

Cherednikov and Batkaev first used Gelevinum for endoscopic prevention of recurrent bleedings in 68 patients with Mallory-Weiss syndrome [21, 35, 38, 63].

Cherednikov [55] conducted more detailed comparative analysis of the results of different endoscopic techniques obtained in 354 patients with Mallory-Weiss syndrome who were treated in a specialized center for patients with gastroduodenal bleedings. The analysis has shown that a sustained (FIA-FIB) bleeding requires a combined method: the so-called liquid diathermocoagulation combined with subsequent insufflation of a granular sorbent (Gelevinum, Diavin) into the tear region. Completed (spontaneously stopped) bleeding in the course of diagnostic fibrogastroduodenoscopy at the pre-hospital stage requires medical endoscopy: endoscopic prevention of recurrent bleedings. The author recommends using a combined method of “liquid” diathermocoagulation of a thrombosed vessel followed by the application of granular sorbents for patients with bleedings of the FIIA type (thrombosed vessel). Patients with bleedings of the FIIB (blood clot) and FIIC types need insufflation of a granular sorbent (Gelevinum, Diavin) into the defective area for recurrent bleeding prevention. All this has made it possible to reduce significantly the risk of recurrent hemorrhage. For instance, endoscopic prevention of recurrent bleedings of the FIIA type has allowed to reduce their frequency 7.4 times (from 22.9 to 3.1 %), and of the FIIB type—3.4 times (from 28.1 to 8.3 %). Such improvement of endoscopic hemostasis with the use of granular sorbents for recurrent hemorrhage prevention has made it possible to avoid emergency operations in patients with Mallory-Weiss syndrome [55].

## Conclusions

Many scientists have published a large amount of information on various etiological factors contributing to the formation of bleeding lacerations in the cardioesophageal area, but, in general, there is no full concept of the pathogenesis of Mallory-Weiss syndrome. After all, the basis of this disease is not just a tear in the layers of the gastroesophageal junction but also a syndrome that should be correctly called discontinuous-hemorrhagic. The nature of this syndrome is not only in the appearance of a tear (defect) but also of a bleeding, anemia, and factors contributing to the syndrome development (increased intra-gastric pressure, vomiting, alcohol, stress, rough food, hunger, work and life disturbances, eating disorders, iatrogenic factor, etc.). It is known that today, there are no specific methods of prediction, prevention, and treatment of discontinuous-hemorrhagic (Mallory-Weiss) syndrome, and they are therefore still to be developed.
